# Estimating the carbon emissions from a resource-limited surgical suite in Papua New Guinea: The climate change potential

**DOI:** 10.1016/j.dialog.2023.100108

**Published:** 2023-02-04

**Authors:** Ian Umo, Margaret Pangiau, John Kukiti, Amos Ona, Sipie Tepoka, Kennedy James, Rodger Ikasa

**Affiliations:** a Surgery Department, Alotau Provincial Hospital, Milne Bay Provincial Health Authority, Papua New Guinea; b Anaesthesia Department, Alotau Provincial Hospital, Milne Bay Provincial Health Authority, Papua New Guinea; c Obstetrics and Gynecology Department, Alotau Provincial Hospital, Milne Bay Provincial Health Authority, Papua New Guinea; d Operating Theatre Department, Alotau Provincial Hospital, Milne Bay Provincial Health Authority, Papua New Guinea

**Keywords:** Climate change, Carbon emissions, Surgical Suite, Papua new guinea, Western pacific

## Abstract

**Introduction:**

The upscale of surgical service delivery in low to middle income countries will increase health sector greenhouse gas emissions globally. Understanding surgical greenhouse gas emissions from surgical suite activities can direct decarbonization strategies and achieve local, and global climate change objectives.

**Material and methods:**

A prospective surgical suite carbon foot print study was conducted at the Alotau Provincial Hospital from the 28^th^ March 2022 to the 28^th^ of May 2022.

**Results:**

The total carbon emission for the surgical suite in APH over the study period was 2,665.8 kgCO_2_e. The average carbon emission per surgical case within the boundary of the surgical suite was 8.4 kgCO_2_e. Scope one emissions (anaesthetic gases) accounted for 44.7% (1171.3 kgCO_2_e) of all carbon emissions.

**Conclusion:**

If no action is taken, carbon emissions in the western pacific region will continue to increase from surgical suites. Therefore, proactive efforts to reduce greenhouse gas emissions must be prioritized.

## Highlights

• The main carbon emission from surgical suites were scope one anesthetic gas emissions.
• Green house gas protocol is used to calculate and report carbon emissions from surgical suites.
• Climate change affects the western pacific region.
• Surgical suites in the western pacific region contributes to carbon emissions globally.

## 1 Introduction

Climate change is a global threat to humanity. Since the pre industrial era, the world has emitted over 1.5 trillion tons of carbon dioxide that has led to an increase in the earths average temperature causing climate change and affecting health [1].

The Lancet Commission of Global Surgery recommends the need to increase essential surgical services [2]. Surgical services are carbon intense and contributes to health sector emissions which represents 4.9% of global green-house gas emissions [3]. This is expected to increase with the scale up of essential surgery in low to middle income countries (LMIC’s) like Papua New Guinea (PNG) where there is a need to improve essential surgical services given the average surgical, anesthesia, and obstetric provider density of 1.8 per 100 000 population [4].

PNG is part of the Asia-Pacific region which accounts for 75% of global natural disaster fatalities [5]. If climate change is not addressed effectively, the economic loss in the region would amount to US$1,957 million across all sectors including agriculture, tourism, and health [5,6]. Much of the strategy in obtaining climate change objectives have been directed towards reducing greenhouse gas emissions, and achieving optimal atmospheric carbon levels [6].

Sources of health sector emissions from surgical suits specifically are a result of drugs and consumable production and waste, anaesthetic gas use, energy consumption and waste (liquid and solid wastes) management activities [7]. There have been numerous studies on carbon emissions from surgical suits in high income countries (HIC’s) [[7], [8], [9], [10]]. MacNeill et al reported annual carbon foot prints of three surgical suits to range between 3,218,907 – 5,187,936 kgCO_2_e with anaesthetic gases and energy consumption being the largest sources of greenhouse gas emissions [8]. A similar study by Drew et al reported the climate impact of surgical procedures to range between 6 – 1,007 kgCO_2_e; where anaesthetic gases were also found to be the highest source of carbon emissions [9]. In United Kingdom it was found that incinerating medical waste had the highest carbon footprint compared to other hospital activities [10].

Despite the large body of evidence, there remains no carbon foot print studies done in surgical suits of low to middle income countries (LMIC’s) where the disparity of surgical services are profound [3]. This is particularly important in Papua New Guinea (PNG) where increasing surgical services would increase local carbon emissions. As such the aim of this study was to estimate the carbon foot print of a resource limited surgical suit in Alotau Provincial Hospital (APH) in the Milne Bay Province of PNG.

## 2 Materials and methods

### 2.1 Study design

A surgical suite carbon foot print study was conducted from the 28^th^ March 2022 to the 28^th^ of May 2022.

### 2.2 Study setting

This study was conducted in APH which serves as the only referral hospital in Milne Bay Province of PNG. It is a surgically capable hospital with a 100 bed capacity that provides for surgical, anesthetic, and obstetric care. There is limited subspecialty services and the hospital has one surgical suit measuring 740m^2^ . The surgical suite comprises one waiting bay, two recovery bays, two operating theatres, and a central sterilization supply division.

### 2.3 Carbon foot print accounting

Emissions in kilograms of carbon dioxide equivalents (kgCO_2_e) were calculated using the Global Warming Potential (GWP) according to the Greenhouse gas protocol [11]. According to this protocol, emissions are reported in three scopes [12].

Scope one refers to direct emissions from within the institutional boundary. Scope two accounts for indirect emissions as a result of electricity consumption, and scope three includes all other indirect emissions occurring as a consequence of the organisation’s activities.

Carbon emissions were thus reported within the boundary of the surgical suite [8]. In this study, scope 1 reported on anesthetic gases; scope 2 reported on electricity consumption and, scope 3 reported on waste disposal. All formulas are shown in Table 1.

**Table 1:** Showing scope formulae.

Scope one kgCO_2_e formulae
CO_2_e (kg) per bottle of anesthetic gas = (Density x Volume x GWP) /1000
Total CO_2_e (kg) of anesthetic gas = Total CO_2_e (kg) of Isoflurane + Total CO_2_e (kg) of Halothane
Scope two kgCO_2_e formulae
Indirect electricity emissions= Total electricity consumption of surgical suit (MWh) x CO_2_ emission factor x GWP_100_
Area method for total electricity consumption of surgical suit (MWh)= Total electricity consumption of hospital (MWh) x [(Area of surgical suit / Total area of all buildings in hospital) x 100]
Scope three kgCO_2_e formulae
Medical waste emissions = Catalogued waste weight (Tons) x waste type production emission factor x GWP_100_
kgCO_2_e per case
CO_2_e (kg) per case= Total carbon emissions of scope 1-3 / Number of operations

Density of inhalation agent in g.cm^3^.

Volume in milliliter’s (mls).

GWP: Global warming potential.

### 2.4 Scope one

Anesthetic gases were recorded by the number of bottles used over the study period. The volume , anesthetic gas density, and their respective GWP were then used to calculate the CO_2_ emissions.

### 2.5 Scope two

A location-based method was used to calculate indirect emissions from electricity [11]. Electricity consumption for the hospital was obtained from PNG power (local electrical utility). PNG power uses diesel fuel to generate electrical energy. The electricity consumption for the surgical suite was obtained using the Area Method as there were no submetering of buildings within the hospital [13]. A default diesel emission factor was used to calculate CO_2_ emissions as the local electrical utility could not provide grid intensities.

### 2.6 Scope three

APH has a fully functioning water, sanitation, hygiene and health care waste service (WASH). Staff have been trained to apply WASH FIT practices using existing infastructure in accordance with the PNG National Water and Hygiene Policy (2015-2030) [14].

An audit of upstream waste disposal was done during the study period by two theatre nurses as per their respective shifts. The operating theatre segregates waste using a three bin system for infectious waste, sharps, and general waste. The waste produced within the study boundary were weighed and catalogued for cardboard, average plastic, polypropylene and sharps. The carbon emissions from body tissues were estimated using the sum of all body tissue estimated and the hazardous waste emission factor (1833 kgCO_2_e/ton) [8].

There are no treatment facilities or incinerators. All waste were transported, and landfilled. Laundry was processed separately from study boundary, and so was excluded. Carbon footprints were calculated by applying greenhouse gas life-cycle conversion factors for catalogued waste [15].

### 2.7 Data collections and other variables

Demographic and surgical characteristics were also collected. These include; sex, age, type of case, operation, extra tray use, complications, anesthesia, and anesthetic time.

Sex was reported as male, or female. Type of cases were reported as either emergency, or elective. Operation were all surgical procedures done within the study boundary with, or without anesthesia. Extra tray use was defined as the opening of an extra tray during surgery. Complications was either an event during the course of surgery that prolonged the anesthetic time, or death on the table. Anesthesia was reported as spinal anesthesia, sedation, local anesthesia, neuroleptic, and none. Anesthetic time was recorded in minutes.

### 2.8 Surgical volume

The yearly surgical volume was 1448 cases in 2021. The minimal sample size of surgical cases was obtained using the Yamane formula (n = N/1+N(e)^2^) [16]; where n = sample size, e = margin of era, and N = population Size. Population size (N) was 1448. Margin of era (e) was taken as 0.05. The minimum surgical volume was 313.

### 2.9 Ethics

The study was approved by the Milne Bay Provincial Health Authority Ethical Research Committee.

## 3 Results

316 operations were done during the study period. Female patients comprised 64.4% (n = 204) of all operations. The mean age of persons undergoing surgery within the study boundary was 35.1 years. Of the total operations 31.6% (n = 100) were done under general anesthesia. Operations done are shown in Table 2. Extra trays were opened in 20.9% (n = 66) of cases. The complication rate was 8.2% (n = 26).

**Table 2:** showing characteristics of operations done over the study period at APH from the 28th March 2022 to the 28th of May 2022.

Characteristics	Total n= 316 (%)
Sex	
Male	112(35.4)
Female	204(64.6)
Age group	
<5 years	20(6.3)
5-15 years	16(5.1)
16-25 years	22(7.0)
26-45 years	81(25.6)
>45 years	177(56.0)
Cases	
Elective	130(41.1)
Emergency	186 (58.9)
Operation	
Bilateral tubal ligation	40(12.7)
Small incision cataract surgery/Intra ocular lens	38(12.0)
Excision	32(10.1)
Removal of foreign body/plate and screw/ Kirschner wires/rush pins	25(7.9)
Exploratory laparotomy	24(7.6)
Caesarean section	21(6.6)
Incision and drainage	20(6.3)
Debridement	18(5.7)
Examination under anaesthesia	15(4.7)
Appendectomy	12(3.8)
Total abdominal hysterectomy	8(2.5)
Herniorrhaphy	7(2.2)
Biopsy	6(1.9)
Bur hole and craniotomy/craniectomy	6(1.9)
Dilatation and curettage	5(1.6)
Tracheostomy/decannulation	5(1.6)
External fixation	4(1.3)
Split skin graft	4(1.3)
Cystotomy	3(0.9)
Intravitreal injection	3(0.9)
Olecranon pin insertion	3(0.9)
Tenotomy	3(0.9)
Circumcision	2(0.6)
Open reduction and internal fixation/Kirschner wire insertion	2(0.6)
Above knee amputation	1(0.3)
Arthrotomy	1(0.3)
Below knee amputation	1(0.3)
Change of dressing	1(0.3)
Colostomy	1(0.3)
Feeding gastrostomy	1(0.3)
Total mastectomy	1(0.3)
Ray amputation	1(0.3)
Manipulation under anesthesia and reduction	1(0.3)
Evisceration	1(0.3)
Extra tray	
Yes	66(20.9)
No	250(79.1)
Anaesthesia	
General anaesthesia	100(31.6)
Local anaesthesia	83(26.3)
Sedation	59(18.7)
Spinal anaesthesia	37(11.7)
Neuroleptic	29(9.2)
Regional block	6(1.9)
None	2(0.6)
Complications	
Yes	26(8.2)
No	290(91.8)

The total carbon footprint of anesthetic gases over the study period was 1,171.30 kgCO_2_e [11]. Isoflurane amounted to 98.0% of the total carbon emissions as shown in Table 3.

**Table 3:** showing carbon emissions from anaesthetic gas use over the study period at APH from the 28th March 2022 to the 28th of May 2022.

Anaesthetic gas	Volume used (mls)	kg CO_2_e (%)
Isoflurane	1,500	1,147.50 (98.0)
Halothane	250	23.8 (2.0)
Total		1,171.30

CO_2_e calculated using 100-year Global Warming Potential (GWP_100_) values of 510 for isoflurane and 50 for Halothane [16].

Total energy consumption over the study period for APH was 121.26 MWh. The surgical suite measured 6.2% of the total area of buildings (11 841m^2^) within APH. Electricity consumption for the surgical suit was calculated to be 7.5 MWh over the study period; with an energy intensity of 10.1 kWh/m^2^. The total indirect surgical suite electricity emissions for the study period was 553.8 kgCO_2_e; using the default emission factor of diesel oil of 73.84 kg CO_2_ per mmBtu [17].

The mean daily waste for the surgical suite was 5.7kg. The total carbon emission for surgical suite waste was 940.7 kgCO_2_e as shown in Table 4. APH does not have an incinerator, and all waste were transported, and landfilled. A total of 52.8 gallons of diesel was used for transportation. Using mobile CO_2_ factor for diesel (10.21 kgCO_2_e per gallon), the carbon emission for transport was calculated to be 539.1 kgCO_2_e.

**Table 4:** Showing carbon emission from surgical suite waste over the study period at APH from the 28th March 2022 to the 28th of May 2022.

Waste type	Total weight (tons)	Production emission factors (kg CO_2_e/tons)	Kg C0_2_e (%)
Cardboard	0.0791	1038	82.1 (8.7)
Average plastic	0.0103	3179	32.7 (3.5)
Polypropylene	0.0375	3254	122 (13.0)
Sharps	0.036	3281	118.1 (12.6)
Transport®	-	-	539.1 (57.3)
Body tissue	0.0255	1833	46.7 (4.9)
Total			940.7

Waste type emission factors obtained from study by MacNeill et al^12^.Sharp emission factor obtained from Cheetham and Johnson: https://network.sustainablehealthcare.org.uk/sites/default/files/resources/syringes_case_study_0_0.pdf

®Calculations for kg CO_2_ e transport was based on mobile CO2 factor for diesel (10.21 kg CO_2_ e per gallon) [17].

The total carbon emission for the surgical suite in APH was 2,665.8 kgCO_2_e (Table 5). Anaesthetic gases accounted for 44.7% (1171.3 kgCO_2_e) of all carbon emissions. The average carbon emission per surgical case within the surgical suite was 8.4 kgCO_2_e.

**Table 5:** showing the scope emissions of the surgical suite of APH from the 28th March 2022 to the 28th of May 2022.

	kgC0_2_e (%)
Scope one	1171.3 (43.9)
Scope two	553.8 (20.8)
Scope three	940.7 (35.3)
Total	2,665.8

## 4 Discussion

The upscale of surgical service delivery will increase global greenhouse gas emissions from operating theaters. The burden is poorly understood in LMIC’s and this study investigates the impact a resource limited surgical suite would have on carbon emissions in order to guide local mitigation, and adaption practices.

Anaesthetic gases are potent contributors to global warming despite their medical necessity. Scope one emissions were found to be the highest emitters of carbon dioxide in this study. Though significant, the emissions were less compared to operating theatres in high income countries (HIC’s) that emit up to 2.1 billion tons of CO_2_e per year [2].

Isoflurane and Halothane were the only two inhalation gases used when compared to other modern anaesthetic gases [18]. Desflurane in particularly has the highest GWP of 2540 [19]. In 2014, 80% of the 3 million tons of carbon dioxide emitted from inhalation gases were from desflurane alone [20]. The high costs of desflurane, and other volatile inhalation gases limits its use in LMIC’s [21]. Cheaper and less potent gases like Isoflurane have much lower GWPs, and studies have reported no difference in patient outcomes [22,23].

The major type of energy consumed in hospitals is electricity [24]. In PNG, 37.3% of electricity is diesel generated, and is the only source of energy in this study [25]. Health care facilities are responsible for 10.3% of the total energy consumption within the building sector [26]. This high energy consumption is directly proportional to space; that requires heating, cooling, ventilation, and lighting [26].

In the current study, the energy intensity was 10.1 kWh/m^2^. This is significantly lower than the average energy intensity of an operating theatre in Germany (36 kWh/m^2^) [27]. Regardless of geography, the overall energy consumption contributes to greenhouse gas emissions globally.

Operating theatres account for one third of all hospital waste with emissions from HICs reported to be as high as 650,436 kgC0_2_e [2,28]. In this study, emissions from operative wastes were 600 times lower. The disparity however is a result of differences in surgical volume, surgeries, waste type, waste segregation, and disposal.

In practicality, scope three emissions were underestimated. MacNeill et al reported that using production emission factors for surgical consumables (as in this study) only reflects emissions embedded in the raw materials and does not capture emissions involved in the manufacturing, sterilisation, and transport of end products [2]. This would have resulted in higher scope three emissions.

In APH, operative waste were landfilled without incineration, or treatment. Rizan et al reported incineration of medical waste to have the highest carbon footprint in hospitals, producing up to 1074 kgCO_2_e/t [10]. Furthermore, the improper waste disposal reported in this study is of local concern. The environmental impact this has can lead to generation of carcinogens like dioxin and leaching of heavy metals, and radioactive substances into the surrounding environment [29]. Landfilling of untreated body tissues can also lead to spread of infectious disease [30]. As such it is imperative that safe treatment and disposal of infectious materials must be made a priority in all hospital throughout PNG for the benefit of human health, the environment and the ecosystem in general.

This study is not without its limitations. Due to COVID-19 and drugs and consumable shortages at APH, only emergency cases were done from the end of May (2022) onwards. This resulted in fewer operations and possible underreporting of carbon emissions. The limited availability of local emission factors, sanitation reporting and lack of submetering made less accurate calculations of carbon dioxide emissions in this study. In addition, reusable consumables like laryngeal masks, infectious waste such as contaminated swabs and other general waste such as food remains were not captured during the study period.

Spilker reports that 90% of anaesthetic emissions can be reduced by adapting greener anaesthetic practices [31]. Reduction of scope one emissions can thus be achieved by minimizing the use of volatile anaesthetic gases, using low flow anaesthesia, using regional anaesthesia where possible, improving close circuit delivery of anaesthesia, and installing an anaesthesia gas scavenging system [7,31].

In PNG there is a potential to utilize renewable forms of energy [25]. However, it is imperative that proper energy auditing through submetering of buildings and equipment is needed to guide reduction strategies and practices. Furthermore, the use of recyclable, reusable and biodegradable medical consumables can reduce emissions [32]. Simple surgical practices like proper packing of surgical trays to reduce unnecessary opening of extra trays during surgery can save energy, and reduce medical waste.

There is a need to integrate climate change health policies in PNG. This can be done by appropriate awareness and training of health workers on mitigation, and adaptation practices within the surgical suit, and health facilities. More broadly, it is imperative that we align our national surgical, obstetric, and anaesthesia plans to incorporate carbon reduction strategies that target infastructure, service delivery, workforce, information systems, financing, and governance [7].

## 5 Conclusion

If no action is taken, carbon emissions in the western pacific region will continue to increase from surgical suites. Therefore, we must not play victim to climate change but rather be proactive in our efforts to reduce greenhouse gas emissions globally.

## Declaration of Competing Interest

All authors hereby declare that there are no conflict of interests.

## Appendix A Supplementary data

Calculation method for anaesthetic gas carbon emissions. Image 1

Calculation method for carbon emissions for power usage. Image 2

Raw data on operating theatre waste Image 3
