# Supplementary material for: Estimating the carbon emissions from a resource-limited surgical suite in Papua New Guinea: The climate change potential
Source: Dialogues Health. 2023 Feb 4;2:100108. doi: 10.1016/j.dialog.2023.100108 (PMC10953991; doi:10.1016/j.dialog.2023.100108)
Supplement: Supplementary file 1 — Calculation method for anaesthetic gas carbon emissions. [file mmc1.docx]

**Anesthetic gas CO2e calculation formula**

CO2e (kg) per bottle of inhalation gas = (Densitiy x Volume x GWP) /1000

**Where:**

Density: density of inhalation agent in g.cm-3 (same g/ml)

Volume: In mls

GWP (Global warming potential)

Isoflurane GWP=510

Halothane=50

Isoflurane density=1.5

Halothane density=1.9

**Total sum**

Total CO2e (kg) of anesthetic drugs = Total CO2e (kg) of X + Total CO2e (kg) of X + Total CO2e (kg) of X + Total CO2e (kg) of X …..

I
