# Supplementary material for: Estimating the carbon emissions from a resource-limited surgical suite in Papua New Guinea: The climate change potential
Source: Dialogues Health. 2023 Feb 4;2:100108. doi: 10.1016/j.dialog.2023.100108 (PMC10953991; doi:10.1016/j.dialog.2023.100108)
Supplement: Supplementary file 2 — Calculation method for carbon emissions for power usage. [file mmc2.docx]

Scope 2 calculations

- Calculating scope 2 emissions requires a method of determining the emissions associated with electricity consumption.
- Market based method: The market-based method reflects the GHG emissions associated with the choices a consumer makes regarding its electricity supplier or product.
- Consumers who do not make specified purchases or who do not have access to supplier data should use the residual mix emission factor to calculate their market-based total.
- In the case of supplier-specific emission factors, the emission factor should reflect emissions from all delivered energy, not just from generation facilities owned/operated by the utility.
- In results we must report where we receive our power from

Steps:

1. Emmision source: PNG power
2. Identify method: Location based method (following the flow chart on page 45)
3. State activity data and get total Mega watts used for time period of study(. In some cases these may not be available, as with consumption occurring in a shared space without energy metering; As such we will use the “area method”)
4. Obtain emission factor from (IEA national electricity emission factors): See: http://data.iea.org/ieastore/product.asp?dept_

id=101&pf_id=304.


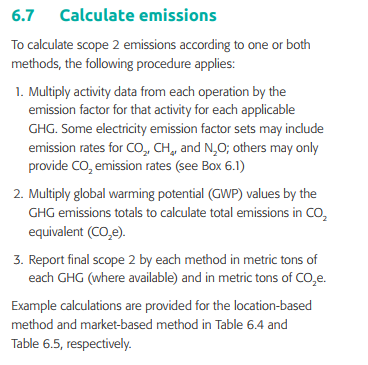


Therefore

Area method to get MWh for OT building

1. Calculate the percentage area of OT building compared to all other buildings in hospital
2. Get total MWh for study period
3. Calculate MWh per building and obtain for OT building

Scope 2 total emissions = MWh x Co2e x GWP

=7.5 x 73.84kg-C02/GJ x 1

=553.8 kg CO2 e

Reference for emission factor : Emission factors.2014.PDF.Available online: https://www.epa.gov/sites/default/files/2015-07/documents/emission-factors_2014.pdf
